# Clinique des Maladies du Système Nerveux

**Published:** 1893-06

**Authors:** 


*Clinique des Maladies du Système Nerveux.* M. le Professeur CHARCOT. Leçons du Professeur, Mémoires, Notes et Observations parus pendant les années 1889-1890, et 1890-91, et publiés sous la direction de GEORGES GUINON, Chef de Clinique. Tome I. Pp. 464. Paris: VEUVE BABÉ ET CIE. 1892.

In this volume are brought together the most important of the observations published by Prof. Charcot in various journals during the period from November, 1889, to November, 1891. Essentially it consists of a series of lectures on neurological subjects, written in that graphic style of which M. Charcot is so acknowledged a master. It is superfluous for us to say that all his writings are characterised by originality and profound powers of thought and observation, and are also conspicuous for ready appreciation of the labours of others.

The first lecture describes the symptoms of Morvan's disease, which is probably a variety of syringomyelia; and in this and a second lecture its relationships to the latter disease, and the differential diagnosis are fully considered, with some further remarks on the anatomical and pathological lesions present. A third chapter gives the account of a case seen by Prof. Charcot in 1875, and again in 1890, after the morbid entity of syringomyelia had been in the interval clearly differentiated.

As we should expect in a volume from the Salpêtrière, several lectures deal with various aspects of hysteria. In one there is a masterly account of hysterical tremor, the varieties of which M. Charcot was the first to describe and analyse. Others deal with traumatic hysteria and the rare "œdème bleu" of hysterical patients.

There is a description of relapsing or periodic oculo-motor paralysis. On account of the concomitant headache, and other symptoms, the author prefers the name of migrainous ophthalmoplegia (*migraine ophthalmoplégique*) for this affection.

Specially worthy of study is the excellent series of articles on the ordinary form of ophthalmoplegia, *externa et interna*, and its relations and connections with the other forms of nuclear degeneration, bulbar paralysis, and general muscular atrophy. We may also mention the chapters on atrophy of the muscles supplied by the peroneal nerve as a complication of severe sciatica, on diabetic paraplegia, on the important subject of cerebral syphilis, and on the equally important one of the aberrant forms of disseminated sclerosis, recently so ably dealt with by Dr. Buzzard. The above will indicate the range of this volume.

